# Overestimation of Severe Acute Respiratory Syndrome Coronavirus 2 Household Transmission in Settings of High Community Transmission: Insights From an Informal Settlement Community in Salvador, Brazil

**DOI:** 10.1093/ofid/ofae065

**Published:** 2024-02-05

**Authors:** Juan P Aguilar Ticona, Nivison Nery, Matt Hitchings, Emilia M M Andrade Belitardo, Mariam O Fofana, Murilo Dorión, Renato Victoriano, Jaqueline S Cruz, Juliet Oliveira Santana, Laise Eduarda Paixão de Moraes, Cristiane W Cardoso, Guilherme S Ribeiro, Mitermayer G Reis, Ricardo Khouri, Federico Costa, Albert I Ko, Derek A T Cummings

**Affiliations:** Instituto de Saúde Coletiva, Universidade Federal da Bahia, Salvador, Bahia, Brazil; Fundação Oswaldo Cruz, Instituto Gonçalo Moniz, Ministério da Saúde, Salvador, Bahia, Brazil; Department of Epidemiology of Microbial Diseases, Yale School of Public Health, New Haven, Connecticut, USA; Instituto de Saúde Coletiva, Universidade Federal da Bahia, Salvador, Bahia, Brazil; Fundação Oswaldo Cruz, Instituto Gonçalo Moniz, Ministério da Saúde, Salvador, Bahia, Brazil; Department of Epidemiology of Microbial Diseases, Yale School of Public Health, New Haven, Connecticut, USA; Department of Biostatistics, University of Florida, Gainesville, Florida, USA; Emerging Pathogens Institute, University of Florida, Gainesville, Florida, USA; Fundação Oswaldo Cruz, Instituto Gonçalo Moniz, Ministério da Saúde, Salvador, Bahia, Brazil; Department of Epidemiology of Microbial Diseases, Yale School of Public Health, New Haven, Connecticut, USA; Department of Epidemiology of Microbial Diseases, Yale School of Public Health, New Haven, Connecticut, USA; Fundação Oswaldo Cruz, Instituto Gonçalo Moniz, Ministério da Saúde, Salvador, Bahia, Brazil; Fundação Oswaldo Cruz, Instituto Gonçalo Moniz, Ministério da Saúde, Salvador, Bahia, Brazil; Fundação Oswaldo Cruz, Instituto Gonçalo Moniz, Ministério da Saúde, Salvador, Bahia, Brazil; Fundação Oswaldo Cruz, Instituto Gonçalo Moniz, Ministério da Saúde, Salvador, Bahia, Brazil; Fundação Oswaldo Cruz, Instituto Gonçalo Moniz, Ministério da Saúde, Salvador, Bahia, Brazil; Centro de Informações Estratégicas de Vigilância em Saúde (CIEVS), Secretaria Municipal de Saúde de Salvador, Salvador, Brazil; Fundação Oswaldo Cruz, Instituto Gonçalo Moniz, Ministério da Saúde, Salvador, Bahia, Brazil; Faculdade de Medicina da Bahia, Universidade Federal da Bahia, Salvador, Bahia, Brazil; Fundação Oswaldo Cruz, Instituto Gonçalo Moniz, Ministério da Saúde, Salvador, Bahia, Brazil; Department of Epidemiology of Microbial Diseases, Yale School of Public Health, New Haven, Connecticut, USA; Faculdade de Medicina da Bahia, Universidade Federal da Bahia, Salvador, Bahia, Brazil; Fundação Oswaldo Cruz, Instituto Gonçalo Moniz, Ministério da Saúde, Salvador, Bahia, Brazil; Instituto de Saúde Coletiva, Universidade Federal da Bahia, Salvador, Bahia, Brazil; Fundação Oswaldo Cruz, Instituto Gonçalo Moniz, Ministério da Saúde, Salvador, Bahia, Brazil; Department of Epidemiology of Microbial Diseases, Yale School of Public Health, New Haven, Connecticut, USA; Fundação Oswaldo Cruz, Instituto Gonçalo Moniz, Ministério da Saúde, Salvador, Bahia, Brazil; Department of Epidemiology of Microbial Diseases, Yale School of Public Health, New Haven, Connecticut, USA; Emerging Pathogens Institute, University of Florida, Gainesville, Florida, USA; Department of Biology, University of Florida, Gainesville, Florida, USA

**Keywords:** BA.1, household transmission, Omicron, SARS-CoV-2

## Abstract

**Background:**

The severe acute respiratory syndrome coronavirus 2 (SARS-CoV-2) Omicron variant has spread globally. However, the contribution of community versus household transmission to the overall risk of infection remains unclear.

**Methods:**

Between November 2021 and March 2022, we conducted an active case-finding study in an urban informal settlement with biweekly visits across 1174 households with 3364 residents. Individuals displaying coronavirus disease 2019 (COVID-19)–related symptoms were identified, interviewed along with household contacts, and defined as index and secondary cases based on reverse-transcription polymerase chain reaction (RT-PCR) and symptom onset.

**Results:**

In 61 households, we detected a total of 94 RT-PCR–positive cases. Of 69 sequenced samples, 67 cases (97.1%) were attributed to the Omicron BA.1* variant. Among 35 of their households, the secondary attack rate was 50.0% (95% confidence interval [CI], 37.0%–63.0%). Women (relative risk [RR], 1.6 [95% CI, .9–2.7]), older individuals (median difference, 15 [95% CI, 2–21] years), and those reporting symptoms (RR, 1.73 [95% CI, 1.0–3.0]) had a significantly increased risk for SARS-CoV-2 secondary infection. Genomic analysis revealed substantial acquisition of viruses from the community even among households with other SARS-CoV-2 infections. After excluding community acquisition, we estimated a household secondary attack rate of 24.2% (95% CI, 11.9%–40.9%).

**Conclusions:**

These findings underscore the ongoing risk of community acquisition of SARS-CoV-2 among households with current infections. The observed high attack rate necessitates swift booster vaccination, rapid testing availability, and therapeutic options to mitigate the severe outcomes of COVID-19.

## Teaser

We found a high level of transmissibility of the SARS-CoV-2 BA.1* Omicron variant both within the community and households. Phylogenetic analysis suggests a diverse set of viruses were transmitted within the community and households, consistent with multiple introductions and high rates of incidence.

The emergence of new severe acute respiratory syndrome coronavirus 2 (SARS-CoV-2) variants poses a significant challenge to public health efforts to control the pandemic. Although the Omicron variant of concern has been linked to lower disease severity [1–3], it has exhibited an unprecedented degree of immune escape, resulting in a high burden of infection even among populations with prior infections and high vaccination coverage [3–6]. Furthermore, the rapid spread of the Omicron variant suggests that it is more transmissible than previous variants, which has important implications for public health control measures [7]. For example, in densely populated settings like informal urban settlements, there is a possibility of a high proportion of secondary infections within households once 1 resident is infected. This situation could diminish the effectiveness of home isolation as a method of controlling transmission in these settings without proper planning.

Previous studies have estimated the household secondary attack rate (SAR) of the BA.1 and BA.2 Omicron variant as ranging from 25% to 81% [4, 8–10]. However, it remains unclear to what extent multiple infections within a household are driven by transmission within the household versus high transmission in the community. Distinguishing household from community-based transmission can be particularly difficult in large outbreaks that spread rapidly among communities as cases both within and between households are clustered in time. Understanding the relative contributions of household and community transmission is crucial for providing appropriate recommendations for infection control. Therefore, we conducted a study to estimate the household secondary transmission of SARS-CoV-2 during the BA.1 Omicron wave (December 2021 to March 2022) in an urban informal settlement in Brazil. We conducted active case-finding of cases within an existing cohort and next-generation sequencing (NGS) analysis to determine if pairs of cases within households and the community were consistent with transmission.

## METHODS

### Study Setting, Design, and Participants

**Figure 1.**
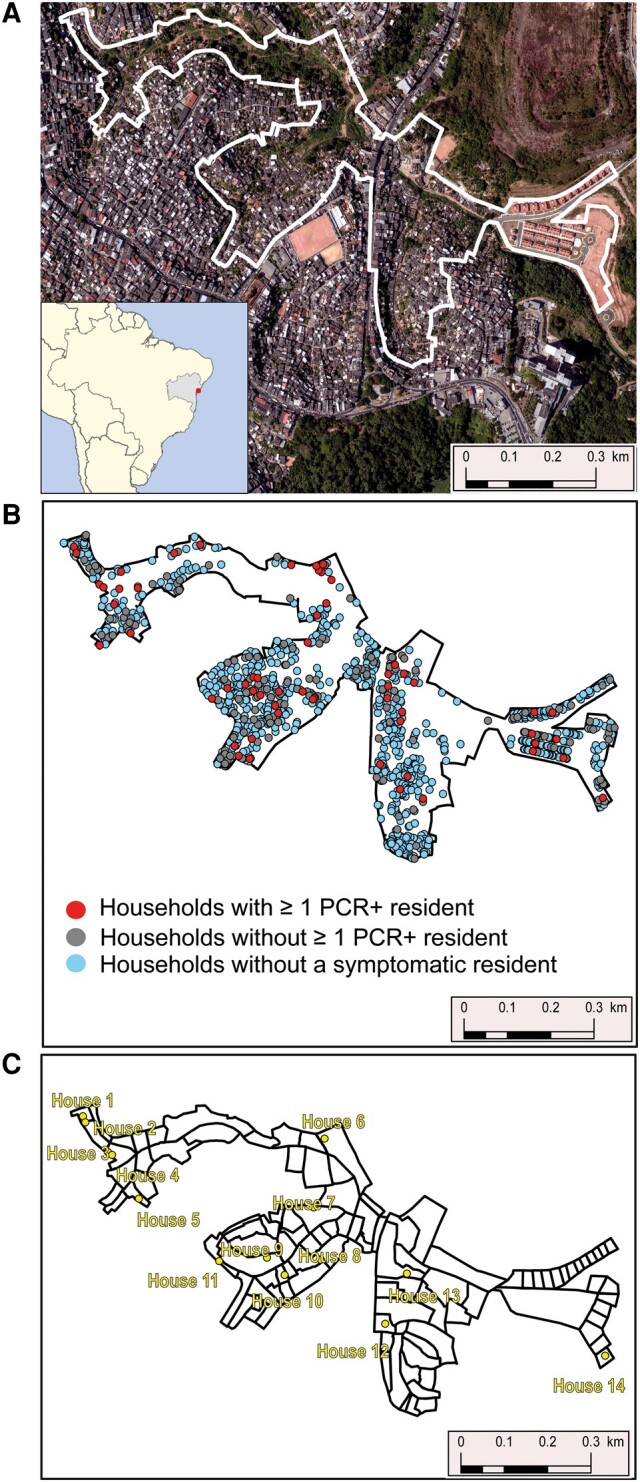
Study setting. *A*, Image of the study area, with inset depicting the location of Salvador and Bahia state within Brazil. *B*, Location of households in the study area with no symptomatic resident (blue dots), no polymerase chain reaction–positive (PCR^+^) resident (gray dots), or at least 1 PCR^+^ resident (red dots). *C*, Fourteen households with >1 resident included in the phylogenetic analysis (yellow dots).

**Figure 2.**
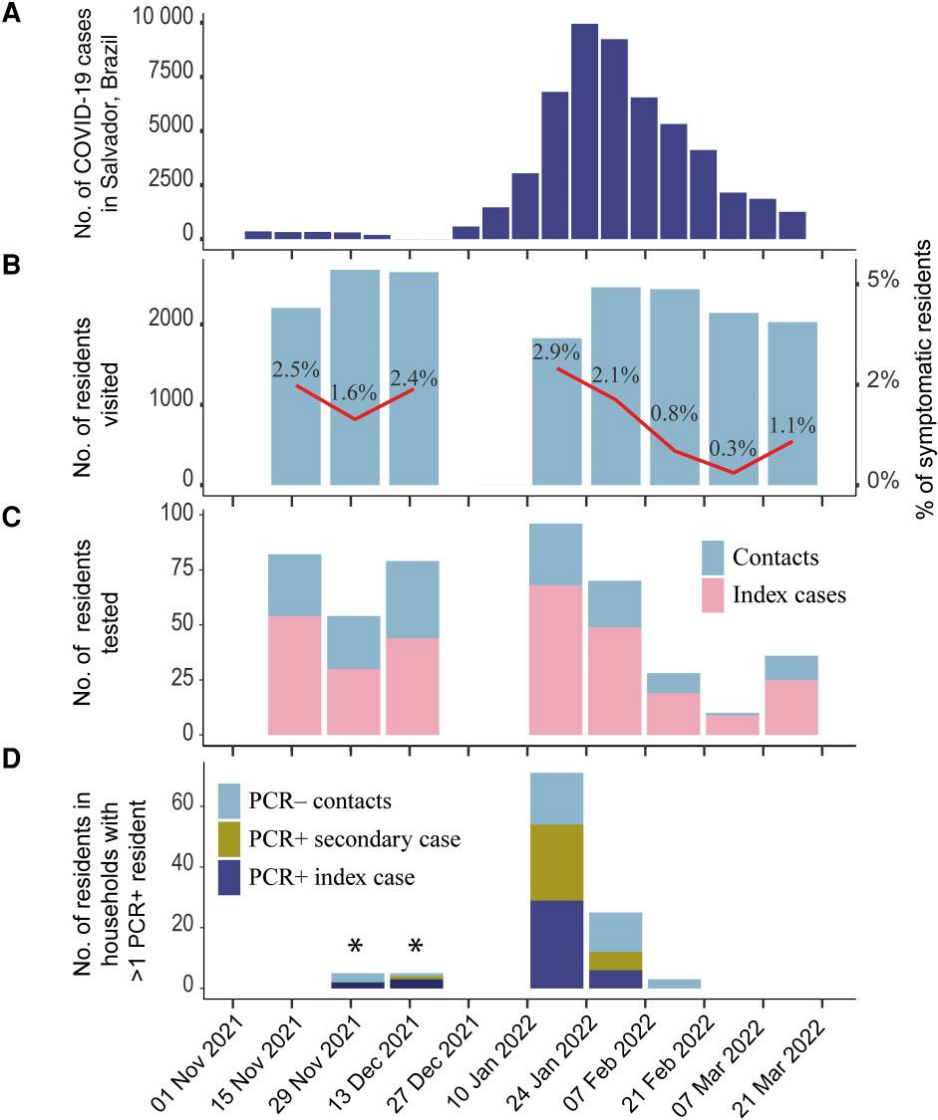
Study period and weekly new cases of coronavirus disease 2019 in Salvador, Brazil (*A*); number of participants screened and proportion with symptoms (*B*); number of participants tested classified as contacts and symptomatic index cases (*C*); and number of participants in households with >1 polymerase chain reaction–positive (PCR^+^) resident (*D*). *No Omicron variants were detected in November and December 2021. Only 2 Delta cases were confirmed; these PCR^+^ results were not included in the secondary attack rate analysis. Abbreviations: COVID-19, coronavirus disease 2019; PCR^–^, polymerase chain reaction negative; PCR^+^, polymerase chain reaction positive.

We conducted this study as a part of an ongoing cohort study in Pau da Lima, an urban informal settlement (in Brazil, commonly called favela) situated in Salvador, the largest city in the northeast region of Brazil. Major characteristics of the informal settlement area have been described in previous studies [11–14] as well as the high willingness for vaccination and the social determinants of vaccine status [13, 15]. In brief, the study area had 3364 inhabitants residing in 1174 households in an area of 0.35 km^2^ comprised of 3 valleys as identified in a previous census conducted in 2021 (Figure 1*A* and 1*B*, Supplementary Figure 1). In December 2021, there was an increase in coronavirus disease 2019 (COVID-19) cases in Salvador associated with the circulation of the SARS-CoV-2 BA.1* Omicron variant (Figure 2*A*).

From 11 November 2021 to 21 March 2022, trained field technicians visited households in the study area every 2 weeks to identify and recruit eligible participants. During each visit, initially, a standardized questionnaire was administered to the head of the household or any adult in the household to identify any residents showing symptoms associated with COVID-19 and to identify their household contacts. Symptomatic cases were defined as participants who reported fever, cough, general weakness/fatigue, headache, myalgia, sore throat, coryza, dyspnea, anorexia/nausea/vomiting, diarrhea, and/or altered mental status [16]. If any symptomatic resident was identified in the household, the study teams performed an individual interview and collection of anterior nasal swabs for all household members, including those without symptoms. The individual interview aimed to assess sociodemographic characteristics, presence and persistence of symptoms, use of health services, and vaccination status. A second visit was scheduled 7 days after the initial visit to identify newly symptomatic residents and collect a second nasal swab from each household member. Participants were included based on their availability, and multiple attempts, including weekends, were made to limit missing data across the 3 valleys comprising the study area.

### Molecular Analysis

Samples collected from symptomatic and asymptomatic household members were tested by real-time reverse-transcription polymerase chain reaction (RT-PCR) to determine SARS-CoV-2 infection status. Positive RT-PCR samples were then subjected to NGS using the Illumina method to identify any variants of concern and/or variants of interest. Both RT-PCR and NGS were conducted by the COVID-19 platform of FIOCRUZ-BA in Brazil.

To perform the phylogenetic analysis, we selected Omicron lineage sequences (BA.1*) from study participants with primer coverage >90%. We compared these sequences with sequences from the city of in Salvador that were collected between 15 September 2021 and 21 March 2022, which were stored in the Global Initiative on Sharing All Influenza Data (GISAID) database. We performed a multiple sequence alignment by using the Fast Fourier Transform (MAFFT v7.505) online alignment server. The aligned genomes were ranked based on their similarity. We used FigTree v1.4.4 to draw the tree and color the tips according to the households they belonged to. We inferred a maximum likelihood tree from the resulting alignment using the general time-reversible substitution model. Additionally, we generated 1000 bootstrap replicates using IQ-TREE v2.2.0.3 (see Supplementary Methods for details).

### Case Definitions

We defined an index symptomatic household case as the resident who reported the earliest onset of symptoms among household participants. Co-index cases were among 2 or more household members with symptom onset on the same date. Household contacts were individuals living in the same household as index cases during the 7 days after the onset of symptoms in the index case. After performing the PCR, index and secondary cases were confirmed. Those household contacts who also tested positive for SARS-CoV-2 were then classified as either symptomatic or asymptomatic secondary cases.

### Data Analysis

We analyzed data using R version 4.2.2 (https://www.r-project.org) software. We used medians and interquartile ranges (IQRs) for numeric variables and frequency and proportions for categorical variables. For bivariate analysis, we used the χ^2^ or Fisher test to compare categorical variables and *t* test or Wilcoxon test to compare continuous variables. Finally, we estimated 95% confidence intervals (CIs) and considered a *P* value <.05 significant.

### Data Analysis: Secondary Attack Rate

The SAR was calculated by dividing the number of secondary cases by the total number of nonindex household residents. Household with co-index were excluded in the calculation of the SAR. We then stratified the SAR by age and sex to evaluate the transmission rate in different groups and identify potential risk factors associated with transmission by calculating the relative risk (RR) and the 95% CIs.

### Data Analysis: Genetic Similarity Analysis

To assess transmission of SARS-CoV-2 within households and the community, we used genomes obtained from whole genome sequencing (WGS) analysis of the virus in the areas of Pau da Lima and Salvador. We constructed a genetic dissimilarity matrix and converted it into a similarity matrix using multidimensional scaling (by exponentiating the values) using the smacof package in R software (https://www.rdocumentation.org/packages/smacof/versions/2.1-5). In this matrix, low values reflect dissimilar sequences, while high values reflect a high degree of pairwise genetic similarity (see Supplementary Methods for details).

To determine the threshold for pairwise genetic similarity between participants that was associated with close transmission, we analyzed 3 groups of sequences. The first group consisted of all individuals in the same household who had >1 confirmed case of SARS-CoV-2. We assumed that this group had a high probability of household transmission (Pau da Lima household group). The second group included 1 participant randomly selected from each household, or the only positive case in the household (Pau da Lima nonhousehold group). The third group included confirmed cases from Salvador, from the same time period as our active surveillance. We analyzed the pairwise genetic similarity within the 3 groups, based on their temporal opportunities for transmission. Then we calculated pairwise similarities and plotted the distribution between the groups. We identified a threshold associated with transmission as the level of genetic dissimilarity at which the cumulative distribution functions of pairwise similarities of within household pairs and nonhousehold pairs visually departed from each other. We then plotted the results of the close transmission analysis using a network graph using Gephi software v0.9.1, to identify possible household transmission among participants.

### Ethics and Patient Consent Statements

The study was approved by the Ethics Committee of the Institute of Collective Health of the Federal University of Bahia (35405320.0.1001.5030), the institutional review boards of the Instituto Gonçalo Moniz, Oswaldo Cruz Foundation (Fiocruz), and the Brazilian National Commission for Ethics in Research (CAAE 45217415.4.0000.0040, 35405320.0.1001.5030, and 59889922.6.0000.0040), and the Yale University Human Research Protection Program (no. 2000031554). Adult participants provided a signed informed consent form in the presence of a witness. For participants <18 years of age, the consent of a parent or legal guardian was required for participation in the study. Children aged ≥6 years also provided written assent to study participation.

## RESULTS

We conducted a total of 8 rounds of biweekly household visits, during which 1098 of 1174 households (94%) participated in at least 1 of the visits (Figure 2*B* and 2*C*). In total 56%–85% of the household were visited in each round (Supplementary Table 1). Among these households, 258 (24%) had at least 1 symptomatic resident, and among them, at least 1 positive case for SARS-CoV-2 by PCR was identified in 61 (27%) households (Supplementary Figure 2). In these households, we identified 94 individuals who tested positive for SARS-CoV-2 by RT-PCR, with 83 of them being symptomatic and 11 asymptomatic (Supplementary Figure 2).

NGS analysis was conducted on 69 of the 94 (73.4%) SARS-CoV-2 PCR-positive samples. The Omicron BA.1* variant was detected in 67 (97.1%) cases, all of which were linked to samples collected between January and February 2022 (Figure 2*D*). The remaining 2 cases (2.9%) were identified as the Delta variant and were linked to samples collected in December 2021.

To evaluate the SAR, we selected a subsample of 35 households with 2 or more residents and with at least 1 documented case of Omicron BA.1*. Households with residents who were infected with Delta variant and households without a confirmed PCR index case or co-index cases were excluded (Supplementary Figure 2). In total, we identified 35 index cases, 31 secondary cases, and 31 contacts that were negative for SARS-CoV-2 among these households. no cases were detected on day 14 visit or later. The crude household SAR was 50.0% (95% CI, 37.8%–62.2%). Individuals aged between 36 and 60 years and females showed a higher SAR and risk ratio than younger individuals (≤18 years old) and males (Table 1).

**Table 1.** Crude Household Secondary Attack Rate With Omicron BA.1 Variant, by Age and Sex

Characteristic	Secondary Cases/Total No. of Contacts	Secondary Attack Rate (95% CI)	RR (95% CI)
Crude SAR	31/62	50.0% (37.0%–63.0%)	…
SAR confirmed by genomic similarity	8/25	32% (13.7%–50.3%)	…
Age group, y			
≤18	8/20	40.0% (21.8%–61.3%)	Ref
19–35	6/19	31.6% (15.4%–54.0%)	0.74 (.30–1.83)
36–60	15/20	75.0% (53.1%–88.8%)	1.82 (1.00–3.30)
≥61	2/3	66.7% (20.7%–93.8%)	1.67 (.64–4.37)
Sex			
Female	20/33	60.6% (43.7%–75.3%)	1.60 (.93–2.74)
Male	11/29	37.9% (22.6%–56.0%)	Ref

Abbreviations: CI, confidence interval; RR, relative risk; SAR, secondary attack rate.

A description of the contacts recruited is presented in Table 2. Among 62 contacts, 50 (80.7%) received at least 1 COVID-19 vaccine dose, similar to the participants in the major cohort (Supplementary Table 2). Of the 40 individuals who participated in the major cohort study and had documented previous exposure, 35 (87.5%) presented a positive immunoglobulin G test result. The comparison between 31 PCR-positive (secondary cases) and 31 PCR-negative household contacts revealed that individuals with secondary SARS-CoV-2 infection were more frequently female (20/31 [64.5%] female vs 11/31 [35.5%] male; RR, 1.6 [95% CI, .9–2.7]) and older (median age, 37 [IQR, 20–43] years vs 22 [IQR, 15–31] years; median difference, 15 [95% CI, 2–21]) than negative contacts (Table 2). However, the risk of secondary transmission did not vary based on vaccination status, prior infection nor other household-level factors (Table 2 and Supplementary Table 3).

**Table 2.** Risk Factors Associated With Household Secondary Transmission of Omicron BA.1 Variant

Characteristics	SARS-CoV-2–Positive Household Contacts	SARS-CoV-2–Negative Household Contacts	RR (95% CI) or Median Difference (95% CI)
	(n = 31)	(n = 31)	
Sex			
Female	20 (64.5)	13 (41.9)	1.60 (.93–2.74)
Male	11 (35.5)	18 (58.1)	Ref
Median age, y (IQR)	37.0 (20–43)	22.0 (15–31.0)	15.0 (2.0–21.0)
Age group, y			
≤18	8 (25.8)	12 (38.7)	Ref
19–35	5 (16.1)	12 (38.7)	0.74 (.30–1.83)
36–60	16 (51.6)	6 (19.4)	1.82 (1.00–3.30)
≥61	2 (6.4)	1 (3.2)	1.67 (.64–4.37)
Reported symptoms			
Symptomatic	21 (67.7)	13 (41.9)	1.73 (1.00–3.04)
Asymptomatic	10 (32.3)	18 (58.1)	Ref
Vaccination status^a^			
Vaccinated	25 (80.6)	25 (80.6)	1 (.53–1.88)
Nonvaccinated	6 (19.4)	6 (19.4)	Ref
Prior SARS-CoV-2 exposure^b^			
Prior exposure	10 (62.5)	11 (68.8)	0.63 (.31–1.31)
No prior exposure	3 (18.8)	1 (6.3)	Ref
Prior SARS-CoV-2 exposure and vaccination status			
Prior exposure and vaccinated	3 (18.8)	2 (12.5)	Ref
Prior exposure and unvaccinated	0	2 (12.5)	0.33 (.03–4.4)
No prior exposure and vaccinated	20 (64.5)	13 (41.9)	1.01 (.47–2.17)
No prior exposure and unvaccinated	11 (35.5)	18 (58.1)	0.63 (.27–1.49)

Data are presented as No. (%) unless otherwise indicated.

Abbreviations: CI, confidence interval; IQR, interquartile range; RR, relative risk; SARS, severe acute respiratory syndrome coronavirus 2.

^a^Individuals who received at least 1 coronavirus disease 2019 vaccine dose.

^b^Positive for SARS-CoV-2 anti-S during previous serosurvey studies in the study site (between July 2021 and September 2022). Only 42 individuals participated in previous serosurveys and had a prior exposure documented.

**Figure 3.**
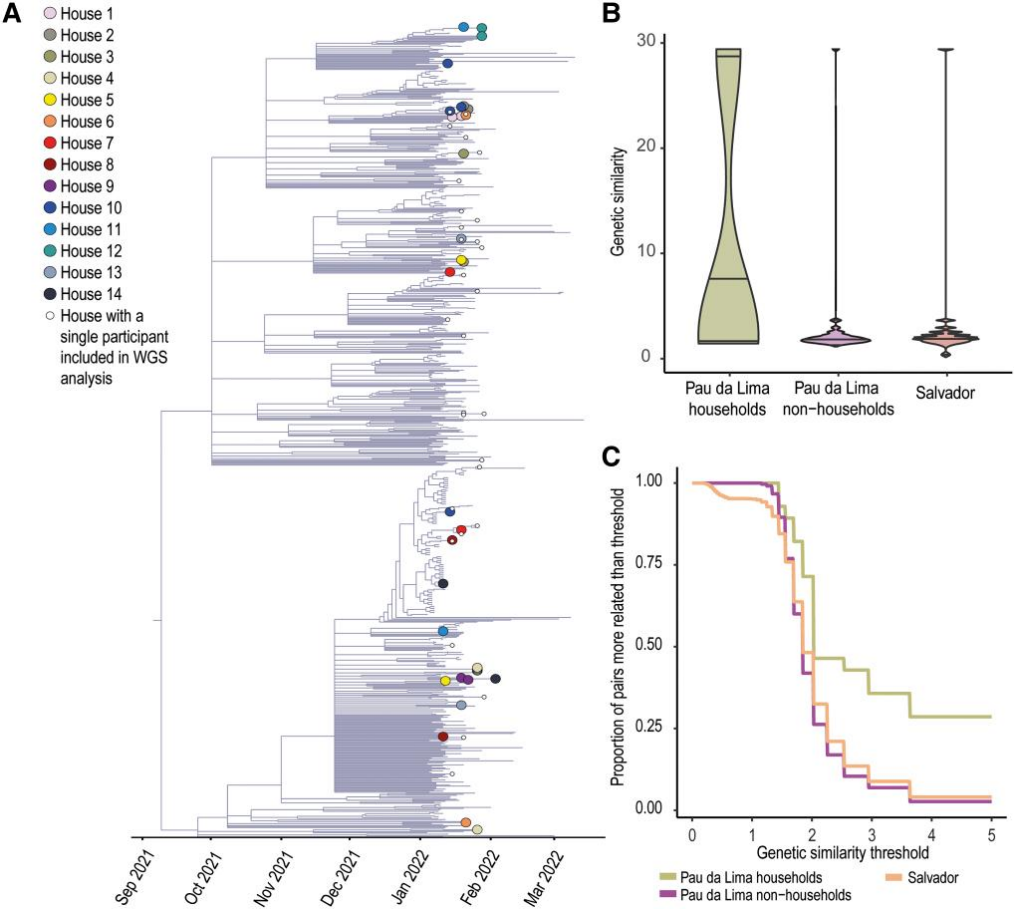
*A*, Time-resolved maximum likelihood phylogenetic tree of the severe acute respiratory syndrome coronavirus 2 variant Omicron BA.1 in Salvador including 62 Omicron BA.1 isolates obtained in this study and an additional 742 representative BA.1 genomes collected throughout the city of Salvador up to 21 March 2022. Colored circles represent participants from 14 households with >1 resident included in the analysis, and small white circles represent households with a single participant. Branches with no circles represent the genomes collected from GISAID. *B*, Genomic similarity among groups. *C*, Proportion of pairs identified at varying genetic similarity thresholds. Abbreviation: WGS, whole genome sequencing.

We included 62 (67.4%) confirmed SARS-CoV-2 Omicron BA.1* sequences in the phylogenetic analysis. This set comprised a subgroup of 33 sequences from 14 households with >1 PCR-positive individual, which allowed us to evaluate the frequency with which household members had virus whose sequence was consistent with transmission between pairs. Furthermore, we identified the presence of single-nucleotide polymorphisms (SNPs) as R346K, I431M, and L450F (Supplementary Figure 3). When comparing the sequences from Pau da Lima to 742 sequences from Salvador, all of them belonged to the same genetic clusters (Figure 3*A*). The similarity metric traversed values of 1 (the largest distance) and a value of 29 as a low distance in pairwise genomic comparisons. The sequences from household pairs in Pau da Lima demonstrated high similarity when compared to sequences from Salvador or the nonhousehold sequences (1 sequence selected per household) from Pau da Lima (Figure 3*B*). In contrast, the genomic similarity between nonhousehold sequences and Salvador city was similar (Figure 3*B*). In brief, this means that there is a notable similarity in sequences among households with 2 or more infected participants when compared to sequences from households with a single infected participant or Pau da Lima or samples from Salvador. This similarity provides evidence supporting household transmission. Finally, we defined a threshold of similarity of ≥2, based on where the cumulative distribution of pairwise differences among pairs departed among household pairs in Pau da Lima compared to nonhousehold pairs and pairs from Salvador (Figure 3*C*).

**Figure 4.**
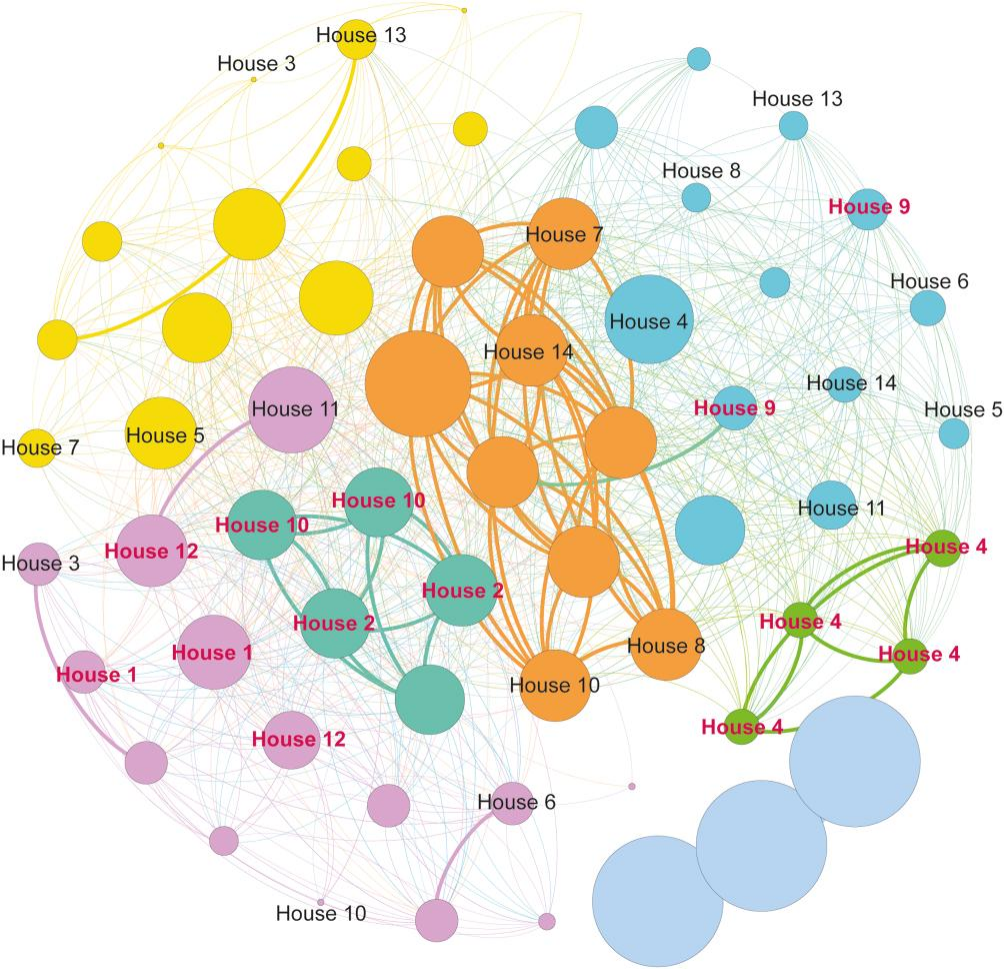
Genetic similarity network of severe acute respiratory syndrome coronavirus 2 (SARS-CoV-2) isolates among study households. Nodes represent individual SARS-CoV-2 sequences and edge weights represent the dissimilarity values between each pair of sequences. The colored nodes on the plot represent sequences from the Pau da Lima community, which are distributed across 6 transmission clusters indicated by the color of the nodes. Sequences with labels belong to households with >1 individual included in this analysis. Red labels indicate potential household transmission based on several household members belonging to the same cluster. The nodes without labels represent sequences from households with a single participant included in the analysis. The lines on the plot indicate genomic similarity (the threshold for genomic similarity is set at >2), with thicker lines representing higher degrees of similarity between sequence pairs. Node size represents the value calculated for betweenness centrality, indicating the amount of influence a node has over the flow of information in the graph [31].

We identified high similarity and interrelation between the viral sequences from this community, leading to the identification of 7 clusters of SARS-CoV-2 community transmission (Figure 4). Within these clusters, we found 14 households with >1 PCR-positive individual and we identified 14 index cases, along with 19 PCR-positive contacts and 14 PCR-negative contacts. However, only 8 secondary transmissions could be confirmed by the similarity analysis as resulting from household transmission. The estimated SAR using the definition based on phylogenetic data was 24.2% (95% CI, 11.9%–40.9%) (Supplementary Figure 4). Finally, we performed a sensitivity analysis comparing households with at least 1 positive PCR against those that reported no symptoms or tested negative. No differences were observed in terms of sex, age, and the mean number of participants <18 years old, demonstrating the representativeness of the participants. However, there was a difference in the number of residents reported by the head of the household, especially in houses with >7 residents (Supplementary Table 4).

## DISCUSSION

Our findings show that the SARS-CoV-2 Omicron variant was highly transmissible in a community that had near-universal previous exposure to SARS-CoV-2 infection and/or vaccination. The household SAR was 50%, and there was no difference in the risk of secondary transmission based on vaccination status or prior infection. During the same period, we compared cases from our community to those from the city of Salvador and determined that rapid transmission and multiple introductions contributed to the high attack rate within our community. Furthermore, we found that a high proportion of infections that were identified as secondary cases in the household investigation could be attributed to community transmission based on the genomic similarity analysis.

Like other informal settlements, Pau da Lima community is characterized by poverty, overcrowding, and poor sanitation [11]. Previous studies in developing countries have highlighted that household overcrowding significantly increases the risk of COVID-19 mortality, primarily affecting older individuals residing in crowded households [17, 18]. Although guidelines suggest maintaining 2 meters of distance among household members and avoiding crowded and inadequately ventilated spaces to limit airborne transmission [19, 20], this is challenging in crowded homes. This scenario is representative of urban formal settlements, as showed by research conducted in India, assessing living conditions in large communities [21]. This research identified overcrowding, unsanitary environments, and restricted access to essential services as primary contributors to the rapid spread of COVID-19 [21]. These structural factors were similar in Pau da Lima where they were associated with a high seroprevalence (48%) during the first wave of SARS-CoV-2 transmission in Brazil [22]. During the initial period of the Omicron wave associated with the BA.1 variant, we observed an elevated household SAR (50%) compared to previous variants [23]. This is in line with the literature, which shows a high transmissibility of the Omicron variant in diverse settings, including high-income countries [24]. Two previous studies in South Korea reported SARs exceeding 50% [25, 26], and a study from the United States reported household transmission ranging from 40.9% among individuals with previous infections to 59.8% among those without [10]. To date, evidence from low- and middle-income countries has been scarce [27]. It is important to determine the main transmission patterns of COVID-19 in communities in order to develop effective preventive strategies. Typically, the household SAR is used to estimate the transmissibility of respiratory viruses such as influenza, but this method may overestimate transmissibility if outside sources of infection are not taken into account [28, 29], particularly if outbreaks in communities are temporally clustered, driving the time scales of household outbreaks and the overall community outbreak to overlap. Our study found evidence of significant community transmission by analyzing the genomic similarities between household members and confirmed cases in the community study site and in Salvador city. By conducting detailed contact tracing and analyzing genomic data, we were able to identify genetically similar viruses within households and better understand transmission patterns. In this analysis, roughly half of putative household transmission pairs were genetically inconsistent with transmission, substantially revising the risk of household acquisition versus community acquisition.

Given the high rate of household transmission and in the community, it may be necessary to recommend additional protective measures, improve ventilation in households, and reevaluate the home isolation during the infectious period in urban informal settlements. Despite the unexpected catastrophic nature of the COVID-19 pandemic, our results emphasize the urgent need for health policies that prioritize equity, especially those supporting urban informal settlements. This demands active engagement from both government and the community as described by Corburn et al [30] and Nix et al [31]. Government and communities should provide support to elevate living standards and upgrade water, sanitation, and hygiene, alongside improved home ventilation, which can also impact other infectious diseases. Community mobilization is also crucial for effective intervention. For instance, community involvement in contact tracing efforts becomes pivotal in identifying potential cases within households and the broader community. Another approach involves immediate and small-scale interventions, such as providing air filters, cooling systems, subsidies for electricity, or access to cooler spaces like community centers. These immediate interventions aim to address the pressing needs and improve conditions swiftly. However, there is a long-term need to address the poor housing conditions in these settlements [31].

The high transmission of the BA.1* Omicron variant observed in our study population emphasizes the level of immune evasion by the new variants and the resulting challenges for transmission control. In our study population, 81% of participants had received at least 1 vaccine dose, and at least 50% had a previous SARS-CoV-2 infection during the first wave of the pandemic in Brazil [11]. These findings are in line with the literature, where the effectiveness of vaccination decreased since the old variants until Omicron [4, 23]. Furthermore, several SNPs identified in the isolates from our study were associated with high immune evasion, including the R346K mutation in the receptor-binding domain, which is associated with weakened neutralizing antibody response [32–34].

In a systematic review of 57 studies, 43 mainly examined the household SAR. The authors of this review indicate that disregarding external sources of infection might lead to an overestimation of SAR within households. The absence of comparisons between secondary and community infections when estimating SAR was acknowledged as a limitation. Also, none of the reviewed studies utilized techniques like WGS to confirm genetic similarity between the strains infecting index and subsequent cases within households [28]. In contrast, our study stands out for its use of phylogenetic analysis, crucial in understanding the community and household transmissions (adjusted SAR, 24.2%) in Pau da Lima, Brazil. Analyzing genetic sequences from individuals in Pau da Lima and Salvador revealed a resemblance between the samples, suggesting multiple virus introductions into this community, making it representative of Salvador city. Despite the absence of clusters in our phylogenetic analysis, our site is representative of the transmission dynamics in Salvador, where 42% of households belong to an urban informal community. Despite limitations in our sequencing scope, we successfully identified transmission clusters within households and the community, highlighting localized virus spread. While acknowledging the need for larger-scale studies to confirm and expand our findings, previous studies utilizing WGS for transmission assessment showed similar outcomes [25, 35].

Our study found that older age and female sex were associated with risk of infection among household contacts. While initial studies conducted prior to the emergence of the Omicron variant showed low prevalence in children and adolescents, as well as low incidence of severe cases and deaths [36], the increased number of infections among children in South Africa [37] and the United Kingdom (UK) [38, 39] during the beginning of the Omicron wave raised concerns for health authorities. A systematic review on SARS-CoV-2 household transmission found a lower secondary transmission to child contacts compared to adults. Interestingly, individuals aged >60 years were identified as the most susceptible to infection [23]. Furthermore, studies in Denmark and the UK observed an increased susceptibility with age and that that the transmission and the SAR were higher for the Omicron variant than previous variants across all age groups [8, 40]. The pattern of household risk may reflect which family members are mostly likely to spend time at home, in contact with other family members and potentially in contact with ill household members. Furthermore, unlike previous COVID-19 waves, the reduction in risk perception, the return to normal activities, and the sense of security following vaccination may have led to an increase in risky behaviors, leaving this population more vulnerable when the Omicron variant emerged. Female participants were also found to be at a higher risk of secondary transmission than male participants, which could be due to social vulnerability factors in urban informal communities [22]. For instance, due to their role as primary family caregivers, women may experience a higher intensity of exposure to infections. This increased exposure can be attributed to factors such as longer duration and closer contact while caring for other sick household members [41, 42].

There are some potential limitations in this study. First, the sample size in this population study was limited, affecting the study's statistical power, as reflected in the wide ranges in the CIs. Second, WGS was not complete for 18 participants with PCR-confirmed infection. However, all these cases were reported between January and February 2022, and the Omicron variant accounted for >95% of the cases in the region during that period; thus, it is plausible that these 18 cases were attributable to the Omicron variant. Third, during the visits, 56%–85% of the households were visited every 2 weeks, based on the availability of the participants. The field team made multiple visits to each household across the 3 valleys comprising the study area, aiming to minimize losses. Finally, the screening protocol was paused from 21 December 2021 to 10 January 2022. It is possible that transmission in the community began during this period and that these early cases were not included in this study.

The high attack rate observed in this study underscores the urgent need to implement prevention measures. This includes reinforcing preventive practices such as handwashing, as well as mask use not only outside the household but also when symptomatic household members are identified. Improving structural housing and health conditions in urban informal settlements (eg, improving ventilation) may also be an important intervention. Our findings demonstrate the need for continued genomic surveillance to not only identify variants and subvariants that represent a hazard to public health, but also for accurate estimation of community and household transmission. Finally, although our results are consistent with existing data on immune evasion of the Omicron variant, it remains crucial to offer booster vaccination and provide access to rapid testing and therapeutics to mitigate the severe outcomes of COVID-19 for vulnerable urban informal residents.

## Supplementary Material

ofae065_Supplementary_Data

## Supplementary Data

Supplementary materials are available at *Open Forum Infectious Diseases* online. Consisting of data provided by the authors to benefit the reader, the posted materials are not copyedited and are the sole responsibility of the authors, so questions or comments should be addressed to the corresponding author.

## Notes

***Acknowledgments.*** The authors thank the residents of the Pau da Lima community in Salvador, Brazil, for their support, and team members from the Collective Health Institute Oswaldo Cruz Foundation, University of Florida, and Yale University.

***Disclaimer.*** The funding sources had no role in the design of this study or its execution, analyses, interpretation of the data, decision to publish, or preparation of the manuscript.

***Financial support.*** This work was supported by the National Institutes of Health (grant numbers R01 AI174105 and R01 AI121207 to A. I. K. and T32AI007517 to M.O.F.); the UK Medical Research Council (grant number MR/T029781/1 to F. C.); the Wellcome Trust (grant numbers 102330/Z/13/Z; 218987/Z/19/Z to F. C.); the Bill & Melinda Gates Foundation (grant number OPP1211988 to M. G. R. and F. C.); the Conselho Nacional de Desenvolvimento Científico e Tecnológico (Brazilian National Council for Scientific and Technological Development, CNPq 311365/2021–3 to G. S. R.); the Fundação de Amparo à Pesquisa do Estado da Bahia (Bahia Foundation for Research Support, FAPESB SUS0019/2021 and PET0022/2016 to G. S. R.); the Burroughs-Wellcome Fund (American Society of Tropical Medicine and Hygiene postdoctoral fellowship to M. O. F.); the William H. Prusoff Foundation (postdoctoral fellowship to M. O. F.); US National Science Foundation (RAPID award to M. D. T. H. and D. A. T. C.); the Coordenação de Aperfeiçoamento de Pessoal de Nível Superior–Brasil (Coordination for the Improvement of Higher Education Personnel; doctoral scholarship to J. P. A. T., finance code 001); the Raj and Indra Nooyi Professorship; the Sendas Family; and Beatrice Kleinberg Neuwirth Funds at the Yale School of Public Health (to A. I. K.).
