# Supplementary material for: Overestimation of Severe Acute Respiratory Syndrome Coronavirus 2 Household Transmission in Settings of High Community Transmission: Insights From an Informal Settlement Community in Salvador, Brazil
Source: Open Forum Infect Dis. 2024 Feb 5;11(3):ofae065. doi: 10.1093/ofid/ofae065 (PMC10957159; doi:10.1093/ofid/ofae065)
Supplement: ofae065_Supplementary_Data [file ofae065_supplementary_data.zip › Supplementary_Fig2.docx]

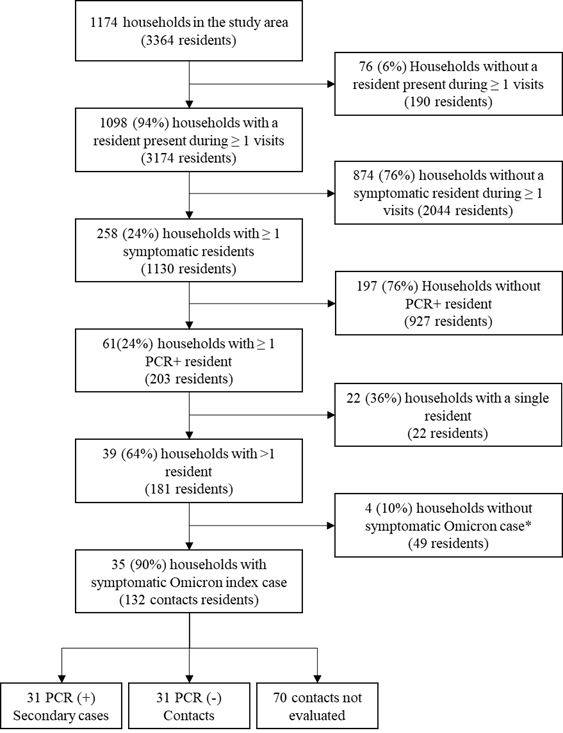


**Supplementary Figure 2.** Study flowchart.

* Four households included two PCR(+) confirmed Delta variants two PCR(+) confirmed secondary cases and one PCR(+) confirmed that did not have an index case defined.
