# Supplementary material for: Overestimation of Severe Acute Respiratory Syndrome Coronavirus 2 Household Transmission in Settings of High Community Transmission: Insights From an Informal Settlement Community in Salvador, Brazil
Source: Open Forum Infect Dis. 2024 Feb 5;11(3):ofae065. doi: 10.1093/ofid/ofae065 (PMC10957159; doi:10.1093/ofid/ofae065)
Supplement: ofae065_Supplementary_Data [file ofae065_supplementary_data.zip › Supplementary_Fig3.docx]

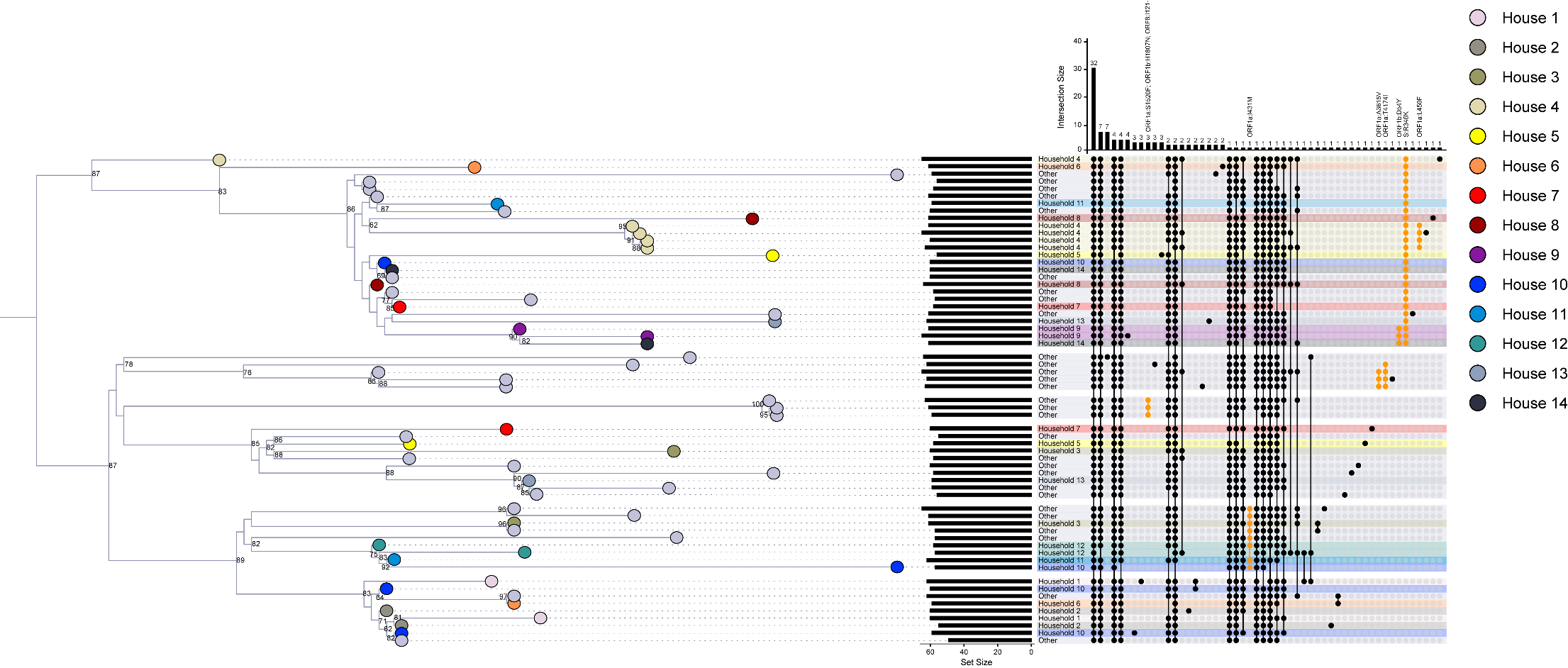


**Supplementary Figure 3.** Phylogenetic tree and SNPs among participants from the community of Pau da Lima. Colored circles in the phylogenetic tree (left side) and colored lines in the matrix (right side) represent households with more than one PCR+ resident. Yellow and black dots represent specific mutations identified, with the yellow dot used to highlight specific clusters among the samples.
