# Supplementary material for: Overestimation of Severe Acute Respiratory Syndrome Coronavirus 2 Household Transmission in Settings of High Community Transmission: Insights From an Informal Settlement Community in Salvador, Brazil
Source: Open Forum Infect Dis. 2024 Feb 5;11(3):ofae065. doi: 10.1093/ofid/ofae065 (PMC10957159; doi:10.1093/ofid/ofae065)
Supplement: ofae065_Supplementary_Data [file ofae065_supplementary_data.zip › Supplementary_methods.docx]

**Household transmission of the SARS-CoV-2 BA.1* Omicron variant in an urban slum settlement in Salvador, Brazil.**

**Supplemental Material**

## Supplemental Methods

## RNA Extraction and RT-qPCR

Samples were extracted from 200 µL of the nasopharyngeal swab eluent using the Quick-DNA/RNA Viral MagBead Kit (Zymo Research, Cat. no. R2141) and the KingFisher Flex System (Thermo Fisher Scientific, Cat. no. 5400630).

SARS-CoV-2 RNA detection was performed by RT-qPCR using the BIOMOL-OneStep/COVID-19 Kit (Instituto de Biologia Molecular do Paraná, ANVISA no. 80780040004), the Molecular SARS-CoV-2 Kit EDx (Bio-Manguinhos, ANVISA no. 80142170045), or the CDC 2019-nCoV Reverse Transcriptase PCR Assay (1) on a 7500 Real-Time PCR System (Applied Biosystems, Cat. no. 4351105) or QuantStudio 5 Real-Time PCR System (Applied Biosystems, Cat. no. A28574). All protocols followed the manufacturer’s instructions.

## NGS Library Preparation and Sequencing

Libraries were prepared using the COVIDSeq Test (Illumina, Cat. no. 20043675 and 20043137) with the ARTIC V4 or V4.1 primer set as they become available. All libraries were pooled together in equimolar amounts. Fragment length distribution was assessed using the Agilent Bioanalyzer High Sensitivity DNA Kit (Agilent Technologies, Cat. no. 5067-4626) on the Agilent 2100 Bioanalyzer (Agilent Technologies, Cat. no. G2939BA). Concentration was assessed using the Qubit 1X dsDNA High Sensitivity Assay Kit (Thermo Fisher Scientific, Cat. no. Q33230 or Q33231) on the Qubit 3 Fluorometer (Thermo Fisher Scientific, Cat. no. Q33216). The library pool was denatured and diluted to a final loading concentration of 8 pM, then loaded into the 300-cycle MiSeq Reagent Kit v2 (Illumina, Cat. no. MS-102-2002) or 600-cycle MiSeq Reagent Kit v3 (Illumina, Cat. no. MS-102-3003). Paired-end sequencing was performed using Illumina MiSeq (Illumina, Cat. no. SY-410-1003) with a 150 bp read length. All protocols followed the manufacturer’s instructions.

## Genome Assembly

The FASTQ files were processed using the pipeline described by Dezordi et al. (2) with minor modifications. Briefly, reads were trimmed to remove low-quality base pairs and primers using fastp v.0.22.0 (3). Assembly was performed by Burrows-Wheeler Aligner (BWA) v.0.7.17 (4) using NCBI GenBank accession no. MN908947.3 as the genome reference. The consensus sequence was then masked with “N” at regions with coverage depth <10, and variant candidates were incorporated into the consensus genome using iVAR v1.3.1 (5). Assembly statistics were calculated with SAMtools v1.15.1 (using HTSlib v1.15.1) (6) and Seqtk v1.3-r106 (<https://github.com/lh3/seqtk>). The sequences generated in this study are available via the GISAID Epi Set identifier EPI_SET_230417xo ([doi: 10.55876/gis8.230417xo](https://doi.org/10.55876/gis8.230417xo)).

## Variant Assignment and Mutation Calling

The lineage assignment was conducted using the Phylogenetic Assignment of Named Global Outbreak Lineages (PANGOLIN) v4.1.2 (7). Mutation calling was performed by Nextclade v2.5.0 (8). The mutation profile was illustrated using an UpSet plot, produced with R v4.2.2 (9) and the following packages: ggplot2 (10), ComplexHeatmap (11), and UpSetR (12). The plot was further processed using Adobe Illustrator CC 2022 (<http://www.adobe.com>).

## Phylogenetic Analysis

We retrieved data for the SARS-CoV-2 BA.1 Omicron variant from Salvador (Northeast Brazil, Bahia) available in the GISAID database (13) between September 15, 2021, and March 21, 2022. To ensure the quality of the data analyzed in this study, only genomes >29,000pb and with a variant assignment provided by the PANGOLIN (7) were considered (n = 742). The complete set of sequences used in the analysis is available via the GISAID Epi Set identifier EPI_SET_230417ns ([doi: 10.55876/gis8.230417ns](https://doi.org/10.55876/gis8.230417ns)). Multiple sequence alignment was performed using MAFFT v7.505 with --6merpair and --addfragments (14,15). The alignment was masked with “N” at all problematic sites (16) and manually inspected using AliView v1.28 (17). The maximum likelihood (ML) phylogenetic analyses were performed using IQ-TREE v2.2.0.3 (18) under the transition model 2 (TIM2) of nucleotide substitution with empirical base frequencies (+F) and a proportion of invariant sites, with 1,000 replicates of ultrafast bootstrapping (--B 1000) and SH-aLRT branch test (--alrt 1000) (19). The best-fitting model was chosen according to the Bayesian Information Criterion inferred by ModelFinder (20) implemented in IQ-TREE. The ML tree topology was transformed into a time-scaled tree using TreeTime v0.9.3 (21). Visualizations of the ML time-scaled tree were produced using R v4.2.2 (9) and the following packages: ggtree (22–24), ggplot2 (10), treeio (25), phangorn (26), readxl (27), svglite (28); and further processed using Adobe Illustrator CC 2022 (<http://www.adobe.com>).

## Genetic Distance Analysis and Network Graph Construction

To evaluate transmission dynamics within households and the community, we constructed a distance matrix using the alignment previously described to allow us to investigate the genetic variations and similarities between all sequences under investigation. The distance matrix parameter settings included terminal gaps and penalized gap-letter matches. Then, we convert the distance matrix into a dissimilarity matrix using the exponential negative transformation method as follows: dissimilarity(i, j) = exp(-distance(i, j)). By applying this transformation, we mapped smaller distances to higher dissimilarity values and larger distances to lower dissimilarity values. The matrixes were produced using R v4.2.2 (9) and the following packages: DECIPHER (29) and smacof (30,31).

We constructed a network graph utilizing the Gephi software v0.9.1. This graph was based on the dissimilarity matrix, with nodes representing the SARS-CoV-2 sequences and edge weights representing the dissimilarity values between corresponding sequences. Self-loops were omitted for clarity, and a threshold-based subgraph was generated, incorporating only edges with weights exceeding 2, predicated on the similarity threshold for transmission. The community structure within the network was determined using modularity analysis (32,33), targeting communities comprising three or more households. The modularity parameters were based on Randomization, edge weights, and a resolution of 0.6. From this analysis, seven distinct communities were identified. To effectively visualize and interpret the graph’s structure, we employed the Fruchterman-Reingold layout algorithm (34) for optimal positioning of vertices. Node size represents the value calculated for betweenness centrality, representing the amount of influence a node has over the flow of information in a graph.

# Reference

1. CDC C for DC and P. CDC 2019-Novel Coronavirus (2019-nCoV) Real-Time RT-PCR Diagnostic Panel [Internet]. Atlanta; 2020. Available from: https://www.fda.gov/media/134922/download

2. Dezordi FZ, Neto AM da S, Campos T de L, Jeronimo PMC, Aksenen CF, Almeida SP, et al. ViralFlow: A Versatile Automated Workflow for SARS-CoV-2 Genome Assembly, Lineage Assignment, Mutations and Intrahost Variant Detection. Viruses. 2022;14(2):217.

3. Chen S, Zhou Y, Chen Y, Gu J. fastp: an ultra-fast all-in-one FASTQ preprocessor. Bioinformatics. 2018;34(17):i884–90.

4. Li H, Durbin R. Fast and accurate long-read alignment with Burrows–Wheeler transform. Bioinformatics. 2010;26(5):589–95.

5. Castellano S, Cestari F, Faglioni G, Tenedini E, Marino M, Artuso L, et al. iVar, an Interpretation-Oriented Tool to Manage the Update and Revision of Variant Annotation and Classification. Genes (Basel). 2021;12(3):384.

6. Danecek P, Bonfield JK, Liddle J, Marshall J, Ohan V, Pollard MO, et al. Twelve years of SAMtools and BCFtools. Gigascience. 2021 Jan 29;10(2):1–4.

7. O’Toole Á, Scher E, Underwood A, Jackson B, Hill V, McCrone JT, et al. Assignment of Epidemiological Lineages in an Emerging Pandemic Using the PangoliO’TOOLE, Áine et al. Assignment of Epidemiological Lineages in an Emerging Pandemic Using the Pangolin Tool. Virus Evolution, [s. l.], v. 7, n. 2, 2021. n Tool. Virus Evol. 2021;7(2).

8. Aksamentov I, Roemer C, Hodcroft E, Neher R. Nextclade: clade assignment, mutation calling and quality control for viral genomes. J Open Source Softw. 2021;6(67):3773.

9. R Core Team. R: A language and environment for statistical computing. R Foundation for Statistical Computing [Internet]. 2016. Available from: https://www.r-project.org

10. Wickham H. ggplot2: Elegant Graphics for Data Analysis. 2nd ed. New York: Springer-Verlag; 2016. 276 p.

11. Gu Z, Eils R, Schlesner M. Complex heatmaps reveal patterns and correlations in multidimensional genomic data. Bioinformatics. 2016;32(18):2847–9.

12. Conway JR, Lex A, Gehlenborg N. UpSetR: An R package for the visualization of intersecting sets and their properties. Bioinformatics. 2017;33(18):2938–40.

13. Khare S, Gurry C, Freitas L, Schultz MB, Bach G, Diallo A, et al. GISAID’s Role in Pandemic Response. China CDC Wkly. 2021;3(49):1049–51.

14. Katoh K, Standley DM. MAFFT Multiple Sequence Alignment Software Version 7: Improvements in Performance and Usability. Mol Biol Evol. 2013;30(4):772–80.

15. Katoh K. MAFFT: a novel method for rapid multiple sequence alignment based on fast Fourier transform. Nucleic Acids Res. 2002;30(14):3059–66.

16. Turakhia Y, De Maio N, Thornlow B, Gozashti L, Lanfear R, Walker CR, et al. Stability of SARS-CoV-2 phylogenies. Barsh GS, editor. PLOS Genet. 2020;16(11):e1009175.

17. Larsson A. AliView: a fast and lightweight alignment viewer and editor for large datasets. Bioinformatics. 2014;30(22):3276–8.

18. Minh BQ, Schmidt HA, Chernomor O, Schrempf D, Woodhams MD, von Haeseler A, et al. IQ-TREE 2: New Models and Efficient Methods for Phylogenetic Inference in the Genomic Era. Mol Biol Evol. 2020;37(5):1530–4.

19. Hoang DT, Chernomor O, von Haeseler A, Minh BQ, Vinh LS. UFBoot2: Improving the Ultrafast Bootstrap Approximation. Mol Biol Evol. 2018;35(2):518–22.

20. Kalyaanamoorthy S, Minh BQ, Wong TKF, von Haeseler A, Jermiin LS. ModelFinder: fast model selection for accurate phylogenetic estimates. Nat Methods. 2017;14(6):587–9.

21. Sagulenko P, Puller V, Neher RA. TreeTime: Maximum-likelihood phylodynamic analysis. Virus Evol. 2018 Jan 1;4(1):1–9.

22. Yu G, Smith DK, Zhu H, Guan Y, Lam TTY. Ggtree: an R Package for Visualization and Annotation of Phylogenetic Trees With Their Covariates and Other Associated Data. Methods Ecol Evol. 2017;8(1):28–36.

23. Yu G, Lam TTY, Zhu H, Guan Y. Two methods for mapping and visualizing associated data on phylogeny using GGTree. Mol Biol Evol. 2018;35(12):3041–3.

24. Yu G. Using ggtree to Visualize Data on Tree‐Like Structures. Curr Protoc Bioinforma. 2020;69(1).

25. Wang L-G, Lam TT-Y, Xu S, Dai Z, Zhou L, Feng T, et al. Treeio: An R Package for Phylogenetic Tree Input and Output with Richly Annotated and Associated Data. Kumar S, editor. Mol Biol Evol. 2020 Feb 1;37(2):599–603.

26. Schliep KP. phangorn: Phylogenetic analysis in R. Bioinformatics. 2011;27(4):592–3.

27. Wickham H, Bryan J. readxl: Read Excel Files [Internet]. 2023. Available from: https://readxl.tidyverse.org

28. Wickham H, Henry L, Pedersen TL, Luciani TJ, Decorde M, Lise V. svglite: An “SVG” Graphics Device [Internet]. 2023. Available from: https://svglite.r-lib.org

29. Wright, Erik S. Using DECIPHER v2.0 to Analyze Big Biological Sequence Data in R. R J. 2016;8(1):352.

30. Leeuw J de, Mair P. Multidimensional Scaling Using Majorization: SMACOF in R. J Stat Softw. 2009;31(3).

31. Mair P, Groenen PJF, de Leeuw J. More on Multidimensional Scaling and Unfolding in R: smacof Version 2. J Stat Softw. 2022;102(10):1–47.

32. Blondel VD, Guillaume J-L, Lambiotte R, Lefebvre E. Fast unfolding of communities in large networks. J Stat Mech Theory Exp. 2008 Oct 9;2008(10):P10008.

33. Lambiotte R, Delvenne J-C, Barahona M. Laplacian Dynamics and Multiscale Modular Structure in Networks. IEEE Trans Netw Sci Eng. 2008;1(2):76–90.

34. Fruchterman TMJ, Reingold EM. Graph drawing by force-directed placement. Softw Pract Exp. 1991 Nov;21(11):1129–64.
