# Supplementary material for: Overestimation of Severe Acute Respiratory Syndrome Coronavirus 2 Household Transmission in Settings of High Community Transmission: Insights From an Informal Settlement Community in Salvador, Brazil
Source: Open Forum Infect Dis. 2024 Feb 5;11(3):ofae065. doi: 10.1093/ofid/ofae065 (PMC10957159; doi:10.1093/ofid/ofae065)
Supplement: ofae065_Supplementary_Data [file ofae065_supplementary_data.zip › Supplementary_Table1.docx]

**Supplementary Table 1. Households and residents visited during the COVID-19 active case finding in the Pau da Lima community.**

| **Round** | **Date** | **Household (HH) visited** | | **number of residents in the household reported by head of the HH** | |
| --- | --- | --- | --- | --- | --- |
|  |  | **(n=1098)** | | **(n=3174)** | |
| 1 | Nov 10 to Nov 23, 2021 | 757 | 68,94% | 2204 | 69,44% |
| 2 | Nov 24 to Dic 07, 2021 | 904 | 82,33% | 2679 | 84,40% |
| 3 | Dic 08 to Dic 21, 2021 | 938 | 85,43% | 2649 | 83,46% |
| break | Dic 22, 2021 to Jan 11, 2022 |  |  |  |  |
| 4 | Jan 11 to Jan 24, 2022 | 616 | 56,10% | 1828 | 57,59% |
| 5 | Jan 25 to Feb 7, 2022 | 852 | 77,60% | 2462 | 77,57% |
| 6 | Feb 8 to Feb 21, 2022 | 831 | 75,68% | 2438 | 76,81% |
| 7 | Feb 22 to Mar 07, 2022 | 738 | 67,21% | 2142 | 67,49% |
| 8 | Mar 08 to Mar 21, 2022 | 705 | 64,21% | 2027 | 63,86% |
