# Supplementary material for: Overestimation of Severe Acute Respiratory Syndrome Coronavirus 2 Household Transmission in Settings of High Community Transmission: Insights From an Informal Settlement Community in Salvador, Brazil
Source: Open Forum Infect Dis. 2024 Feb 5;11(3):ofae065. doi: 10.1093/ofid/ofae065 (PMC10957159; doi:10.1093/ofid/ofae065)
Supplement: ofae065_Supplementary_Data [file ofae065_supplementary_data.zip › Supplementary_Table2.docx]

**Supplementary Table 2: COVID-19 Vaccine coverage in the Pau da Lima Cohort until March 21st, 2022**

| **COVID-19 Vaccine coverage in the Pau da Lima Cohort*** | n (%) |
| --- | --- |
| Vaccination - first dose |  |
| Yes | 1193 (80,6) |
| No | 288 (19,4) |
| Vaccine type |  |
| Pfizer | 597 (50) |
| Coronavac | 322 (27) |
| AstraZeneca - Fiocruz - Oxford | 241 (20,2) |
| Johnson & Johnson | 29 (2,4) |
| NA | 4 (0,3) |
| Vaccination - second dose |  |
| Yes | 989 (66,8) |
| No | 492 (33,2) |
| Vaccine type |  |
| Pfizer | 461 (46,6) |
| Coronavac | 273 (27,6) |
| AstraZeneca - Fiocruz - Oxford | 229 (23,2) |
| Johnson & Johnson | 23 (2,3) |
| NA | 3 (0,3) |
| Vaccination - third dose |  |
| Yes | 346 (23,4) |
| No | 1135 (76,6) |
| Vaccine type |  |
| Pfizer | 234 (67,6) |
| AstraZeneca - Fiocruz - Oxford | 79 (22,8) |
| Coronavac | 11 (3,2) |
| Johnson & Johnson | 16 (4,6) |
| NA | 6 (1,7) |

* Data based on the survey conducted between October 2022 and March 2023 in the cohort of Pua da Lima
