# Supplementary material for: Overestimation of Severe Acute Respiratory Syndrome Coronavirus 2 Household Transmission in Settings of High Community Transmission: Insights From an Informal Settlement Community in Salvador, Brazil
Source: Open Forum Infect Dis. 2024 Feb 5;11(3):ofae065. doi: 10.1093/ofid/ofae065 (PMC10957159; doi:10.1093/ofid/ofae065)
Supplement: ofae065_Supplementary_Data [file ofae065_supplementary_data.zip › Supplementary_Table3.docx]

**Supplementary Table 3.** Household-level factors associated with secondary transmission

|  | **SARS-CoV-2 (+) Household contacts** | **SARS-CoV-2 (-) Household contacts** | p-value |
| --- | --- | --- | --- |
|  | Individual  (N=31) | Individual  (N=31) |  |
| **Number of household contacts** |  |  | 0.128 |
| Median [IQR] | 4.00 [2.50, 4.50] | 3 [2.0 - 4.0] |  |
| **Sex of the index case, n (%)** |  |  | 0.290 |
| Female | 22 (71.0%) | 18 (58.1%) |  |
| Male | 9 (29.0%) | 13 (41.9%) |  |
| **Age groups of the index case, n (%)** |  |  | 0.890 |
| ≤ 18 | 7 (22.6%) | 8 (25.8%) |  |
| 19 - 35 | 8 (25.8%) | 9 (29.0%) |  |
| 36 - 60 | 15 (48.4%) | 11 (35.5%) |  |
| ≥ 61 | 1 (3.2%) | 3 (9.7%) |  |
| **Index case Ct value** |  |  | 0.072 |
| Median [IQR] | 24.3 [21.4 - 26.0] | 25.9 [22.6 - 27.7] |  |
| **Vaccination status of the index case, n (%)** |  |  | 1 |
| Vaccinated | 22 (71.0%) | 22 (71.0%) |  |
| Non-vaccinated | 9 (29.0%) | 9 (29.0%) |  |
