# Supplementary material for: Overestimation of Severe Acute Respiratory Syndrome Coronavirus 2 Household Transmission in Settings of High Community Transmission: Insights From an Informal Settlement Community in Salvador, Brazil
Source: Open Forum Infect Dis. 2024 Feb 5;11(3):ofae065. doi: 10.1093/ofid/ofae065 (PMC10957159; doi:10.1093/ofid/ofae065)
Supplement: ofae065_Supplementary_Data [file ofae065_supplementary_data.zip › Supplementary_Table4.docx]

**Supplementary Table 4.** Comparison of households with ≥ 1 PCR+ resident and households without any PCR+ resident

|  | **Overall** | **Households with ≥ 1 PCR+ resident** | **Households without 1 PCR+ resident*** | **p-value** |
| --- | --- | --- | --- | --- |
|  | **(household = 1014 participants = 2964)** | **(household = 61 participants = 213)** | **(household = 953 participants = 2751)** |  |
| **individual factors** |  |  |  |  |
| **Sex n (%), n = 2961** |  |  |  |  |
| Female | 1644 (55.5%) | 109 (51.2%) | 1535 (55.8%) | 0.242 |
| Male | 1317 (44.4%) | 102 (47.9%) | 1215 (44.2%) |  |
| **Age n (%), n = 2947** |  |  |  |  |
| ≤ 18 | 839 (28.3%) | 56 (26.3%) | 783 (28.5%) | 0.187 |
| 19 – 35 | 963 (32.5%) | 57 (26.8%) | 906 (32.9%) |  |
| 36 – 60 | 934 (31.5%) | 68 (31.9%) | 866 (31.5%) |  |
| ≥ 61 | 211 (7.1%) | 21 (9.9%) | 190 (6.9%) |  |
| **Household factors** |  |  |  |  |
| **Median of No. of residents** | 3.00 [1.0 - 9.0] | 3.00 [1.0 - 7.0] | 3.00 [1.0, 9.0] | <0.001 |
| **Median of No. of residents < 10 years old** | 0 [0, 5.0] | 0 [0, 2.0] | 0 [0, 5.0] | 0.118 |
| **Median of No. of residents between 10 to 17 years old** | 0 [0, 4.0] | 0 [0, 2.0] | 0 [0, 4.0] | 0.692 |

* Data based on the survey conducted between October 2022 and March 2023 in the cohort of Pua da Lima
